# Characteristics of incident substance-induced psychosis compared to incident first-episode psychosis. A nationwide register-linkage study.

**DOI:** 10.1192/j.eurpsy.2023.2026

**Published:** 2023-07-19

**Authors:** J. Jeyaniroshan, P. Sassi

**Affiliations:** 1 Department of psychiatry, University of Turku, Turku, Finland

## Abstract

**Introduction:**

To date, most of the substance-induced psychosis (SIP) risk factor research has focused on meth/amphetamine use with cross-sectional study designs. Register-linkage studies, in turn, have focused mostly on the prognosis of SIP regarding mortality or conversion to schizophrenia. Far less is known about preceding factors before the incident SIP episode such as psychiatric comorbidity or work-related factors.

**Objectives:**

There is no previous research on how persons with SIP differ from persons with other incident psychotic episodes (first-episode psychosis, FEP). This study aims to explore: 1) are their differences in previous psychiatric diagnoses and 2) work-related factors between SIPs and FEPs before the incident psychosis episodes.

**Methods:**

The study covers extensive register-linkage data sets from Sweden. Incident SIP cases (n=7320) were identified from National Patient Register during the years 2006-2016 and matched 1:1 (age, gender, and calendar year) with incident FEP cases. Information from the sociodemographic background, psychiatric disorders, and work-related factors during the proceeding two-year period before the incident SIP/FEP episode were linked from national registries. SIPs vs FEPs were compared using logistic regression analysis, adjusted with education level, family situation, dwelling, country of origin and Charlson Comorbidity Index.

**Results:**

Previous self-harm (OR 2.3;95%CI 2.1-2.6), ADHD (OR 1.8;95%CI 1.6-2.0) and substance dependence diagnoses (OR 7.2;95%CI 6.6-7.9) were more prevalent among SIPs compared to FEPs. In turn, all other previous psychiatric disorder diagnoses were less prevalent among SIPs. Compared to FEPs, SIPs were more often unemployed (OR 1.2;95%CI 1.1-1.2) and had less any employment (OR 0.9, 95%CI 0.9-0.98), but also, they were less often on sickness abstinence over 180 days (OR 1.1, 95%CI 0.9-1.3) The prevalence of previous substance use disorder was most common in alcohol SIP (OR 9.6;95%Cl 7.3-12.7).

**Image:**

**Figure:**
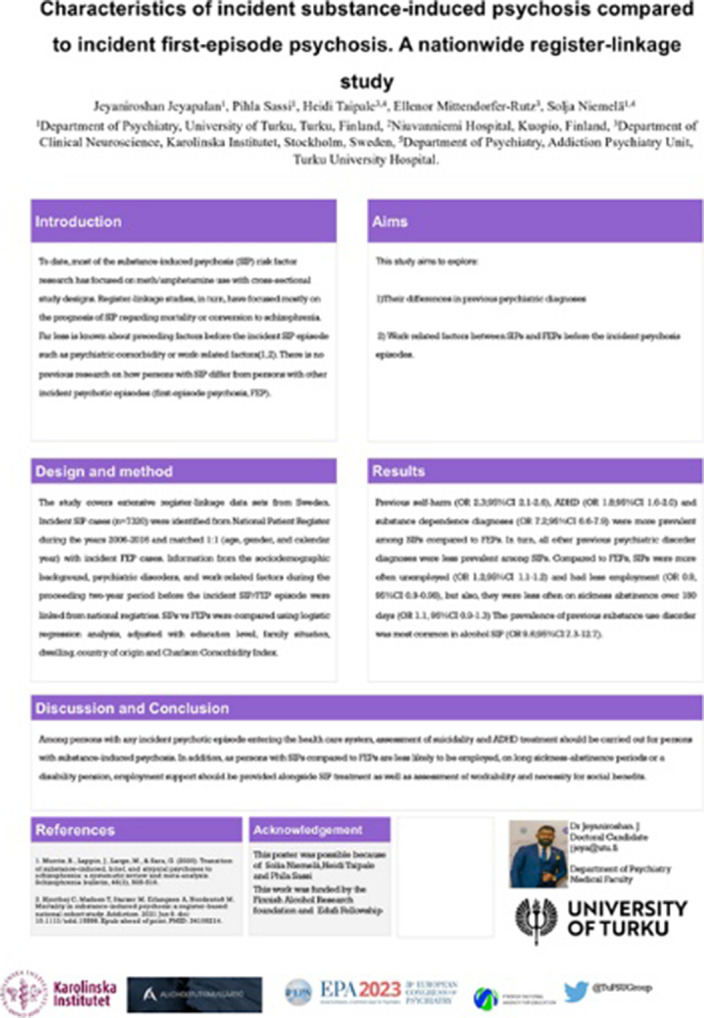

**Conclusions: Discussions and Conclusion:**

Among persons with any incident psychotic episode entering the health care system, assessment of suicidality and ADHD treatment should be carried out for persons with substance-induced psychosis. In addition, as persons with SIPs compared to FEPs are less likely to be employed, on long sickness-abstinence periods or a disability pension, employment support should be provided alongside SIP treatment as well as assessment of workability and necessity for social benefits.

**Disclosure of Interest:**

None Declared

